# Bis(2-amino-1,3-benzothia­zol-3-ium) bis­(7-oxabicyclo­[2.2.1]heptane-2,3-dicarboxyl­ato)cadmate hexa­hydrate

**DOI:** 10.1107/S1600536812022593

**Published:** 2012-05-26

**Authors:** Fan Zhang, Qiu-Yue Lin, Ling-Ling Chen, Jun-Gang Ke

**Affiliations:** a Zhejiang Key Laboratory for Reactive Chemistry on Solid Surfaces, Institute of Physical Chemistry, Zhejiang Normal University, Jinhua, Zhejiang 321004, People’s Republic of China; b College of Chemistry and Life Science, Zhejiang Normal University, Jinhua 321004, Zhejiang, People’s Republic of China

## Abstract

In the structure of the title complex, (C_7_H_7_N_2_S)_2_[Cd(C_8_H_8_O_5_)_2_]·6H_2_O, the Cd^II^ atom is located on an inversion center and is *O*,*O*′,*O*′′-chelated by two symmetry-related 7-oxabicyclo­[2.2.1]heptane-2,3-dicarboxyl­ate ligands in a distorted octa­hedral geometry. The 2-amino­benzothia­zolium cation links with the Cd complex anion *via* N—H⋯O hydrogen bonding. Extensive O—H⋯O and N—H⋯O hydrogen bonds involving lattice water mol­ecules occur in the crystal structure.

## Related literature

For background to the applications of 7-oxabicyclo­[2,2,1]heptane-2,3-dicarb­oxy­lic anhydride (norcantharidin), see: Yin *et al.* (2005 ▶). For a manganese(II) analogue, see: Wang *et al.* (2010*a* ▶), for a cobalt(II) analogue, see: Wang *et al.* (2010*b* ▶), for a nickel(II) analogue, see: Wang *et al.* (2012 ▶) and for a zinc(II) analogue, see: Zhang *et al.* (2012 ▶).
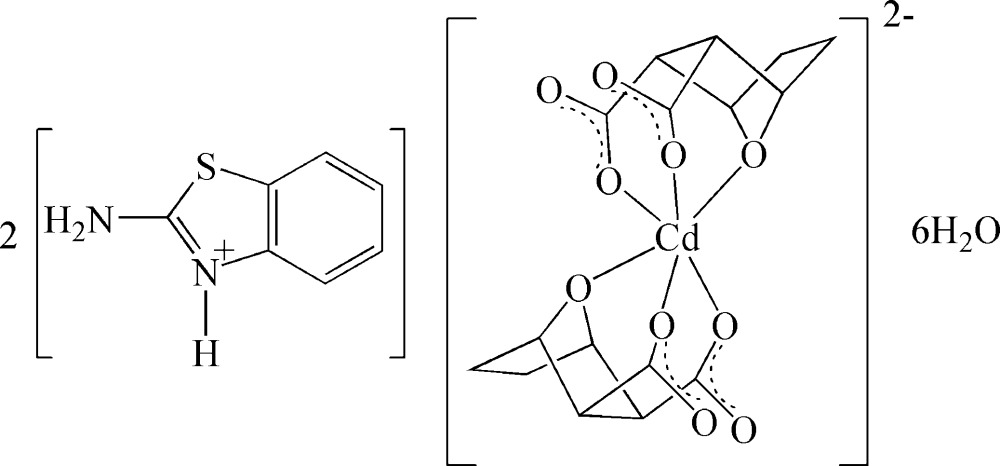


## Experimental

### 

#### Crystal data

(C_7_H_7_N_2_S)_2_[Cd(C_8_H_8_O_5_)_2_]·6H_2_O
*M* *_r_* = 891.23
Triclinic, 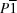

*a* = 6.6990 (8) Å
*b* = 10.3103 (10) Å
*c* = 13.0979 (13) Å
α = 89.039 (7)°
β = 89.004 (7)°
γ = 82.062 (7)°
*V* = 895.76 (16) Å^3^
*Z* = 1
Mo *K*α radiation
μ = 0.81 mm^−1^
*T* = 296 K
0.12 × 0.08 × 0.06 mm

#### Data collection

Bruker APEXII area-detector diffractometer
Absorption correction: multi-scan (*SADABS*; Sheldrick, 1996 ▶) *T*_min_ = 0.927, *T*_max_ = 0.957
12401 measured reflections
3138 independent reflections
2782 reflections with *I* > 2σ(*I*)
*R*_int_ = 0.037

#### Refinement

*R*[*F* ^2^ > 2σ(*F* ^2^)] = 0.029
*wR*(*F* ^2^) = 0.070
*S* = 1.07
3138 reflections
250 parameters
H-atom parameters constrained
Δρ_max_ = 0.39 e Å^−3^
Δρ_min_ = −0.33 e Å^−3^

### 

Data collection: *APEX2* (Bruker, 2006 ▶); cell refinement: *SAINT* (Bruker, 2006 ▶); data reduction: *SAINT*; program(s) used to solve structure: *SHELXS97* (Sheldrick, 2008 ▶); program(s) used to refine structure: *SHELXL97* (Sheldrick, 2008 ▶); molecular graphics: *SHELXTL* (Sheldrick, 2008 ▶); software used to prepare material for publication: *SHELXL97*.

## Supplementary Material

Crystal structure: contains datablock(s) I, global. DOI: 10.1107/S1600536812022593/vn2038sup1.cif

Structure factors: contains datablock(s) I. DOI: 10.1107/S1600536812022593/vn2038Isup2.hkl

Additional supplementary materials:  crystallographic information; 3D view; checkCIF report

## Figures and Tables

**Table 1:** Selected bond lengths (Å)

Cd1—O1	2.2108 (18)
Cd1—O3	2.2954 (18)
Cd1—O5	2.3499 (17)

**Table 2:** Hydrogen-bond geometry (Å, °)

*D*—H⋯*A*	*D*—H	H⋯*A*	*D*⋯*A*	*D*—H⋯*A*
O1*W*—H1*WA*⋯O2	0.85	1.85	2.682 (3)	167
O2*W*—H2*WB*⋯O3*W*	0.85	2.15	2.995 (3)	170
O1*W*—H1*WB*⋯O3*W*	0.85	1.96	2.806 (3)	173
O3*W*—H3*WB*⋯O4	0.85	2.02	2.837 (3)	161
N1—H1*A*⋯O4^i^	0.86	1.84	2.700 (3)	176
N2—H2*A*⋯O3^i^	0.86	2.00	2.845 (3)	169
N2—H2*B*⋯O1*W*^ii^	0.86	2.00	2.824 (3)	160
O2*W*—H2*WA*⋯O1*W*^ii^	0.85	1.93	2.758 (4)	164
O3*W*—H3*WA*⋯O2*W*^iii^	0.85	1.96	2.794 (3)	166

Symmetry codes: (i) 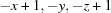; (ii) 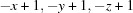; (iii) 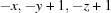.

**Fig. 1.**
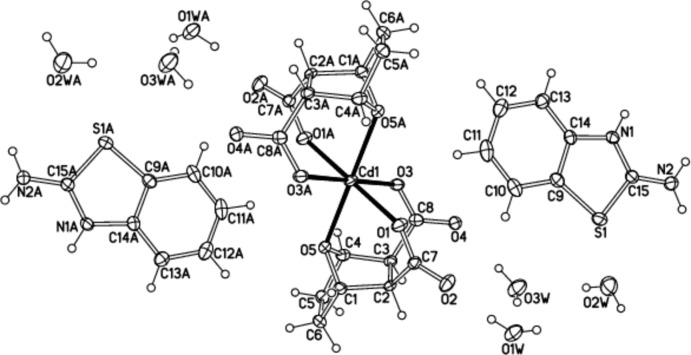
A view of the molecule of (I) showing the atom-labelling scheme with displacement ellipsoids drawn at the 30% probability.

## supplementary crystallographic information

### Comment

7-oxabicyclo[2,2,1]heptane-2,3-dicarboxylic anhydride (norcantharidin), which possesses great anti-cancer activity, has been used in clinic tests (Yin *et al.*, 2005). An isostructural norcantharadin manganese complex (Wang *et al.*, 2010*a*), a cobalt complex (Wang *et al.*, 2010*b*), a nickel complex (Wang *et al.*,2012) and a zinc complex (Zhang *et al.*, 2012) have been reported. The molecular structure of the title complex is shown in Fig.1. The cadmium atom is six-coordinated in a distorted octahedral coordination mode, binding to two bridging O atoms of the bicycloheptane unit and four carboxylate O atoms of two symmetry-related and fully deprotonated ligands. 2-aminobenzothiazole is not involved in the coordination of the cation, and N atom of thiazole ring is protonated.The crystal structure is stabilized by N—H···O hydrogen-bonding interactions between the cations and anions and O—H···O hydrogen bonds including the crystal water molecules.

### Experimental

A mixture of 0.5 mmol norcantharidin, 0.5 mmol cadmium acetate, 0.5 mmol 2-aminobenzothiazole and 15 mL distilled water was sealed in a 25 mL Teflon-lined stainless vessel and heated at 443 K for 3 d, then slowly cooled to room temperature. The solution was filtered and block-shaped colorless crystals were obtained.

### Refinement

The H atoms bonded to O atoms were located in a difference Fourier maps, repositionned to a correct geometry and subsequently refined using a riding model and allowed to rotate around the pivot oxygen atom (AFIX 6 in SHELXL). The isotropic ADP of the water hydrogen atoms were set as follows: *U*_iso_(H) = 1.5*U*_eq_(O). Other H atoms were positioned geometrically and refined using a riding model with C—H = 0.97–0.98 and N—H = 0.86 Å, *U*_iso_(H) = 1.2*U*_eq_(C,N).

### Crystal data

**Table:**

(C_7_H_7_N_2_S)_2_[Cd(C_8_H_8_O_5_)_2_]·6H_2_O	*Z* = 1
*M**_r_* = 891.23	*F*(000) = 458
Triclinic, *P*1	*D*_x_ = 1.652 Mg m^−^^3^
Hall symbol: -P 1	Mo *K*α radiation, λ = 0.71073 Å
*a* = 6.6990 (8) Å	Cell parameters from 3684 reflections
*b* = 10.3103 (10) Å	θ = 1.6–25.0°
*c* = 13.0979 (13) Å	µ = 0.81 mm^−^^1^
α = 89.039 (7)°	*T* = 296 K
β = 89.004 (7)°	Block, colourless
γ = 82.062 (7)°	0.12 × 0.08 × 0.06 mm
*V* = 895.76 (16) Å^3^	

### Data collection

**Table:**

Bruker APEXII area-detector diffractometer	3138 independent reflections
Radiation source: fine-focus sealed tube	2782 reflections with *I* > 2σ(*I*)
Graphite monochromator	*R*_int_ = 0.037
ω scans	θ_max_ = 25.0°, θ_min_ = 1.6°
Absorption correction: multi-scan (*SADABS*; Sheldrick, 1996)	*h* = −7→7
*T*_min_ = 0.927, *T*_max_ = 0.957	*k* = −12→12
12401 measured reflections	*l* = −15→15

### Refinement

**Table:**

Refinement on *F*^2^	Primary atom site location: structure-invariant direct methods
Least-squares matrix: full	Secondary atom site location: difference Fourier map
*R*[*F*^2^ > 2σ(*F*^2^)] = 0.029	Hydrogen site location: inferred from neighbouring sites
*wR*(*F*^2^) = 0.070	H-atom parameters constrained
*S* = 1.07	*w* = 1/[σ^2^(*F*_o_^2^) + (0.0329*P*)^2^ + 0.3371*P*] where *P* = (*F*_o_^2^ + 2*F*_c_^2^)/3
3138 reflections	(Δ/σ)_max_ = 0.001
250 parameters	Δρ_max_ = 0.39 e Å^−^^3^
0 restraints	Δρ_min_ = −0.33 e Å^−^^3^

### Special details

**Table:**

Geometry. All esds (except the esd in the dihedral angle between two l.s. planes) are estimated using the full covariance matrix. The cell esds are taken into account individually in the estimation of esds in distances, angles and torsion angles; correlations between esds in cell parameters are only used when they are defined by crystal symmetry. An approximate (isotropic) treatment of cell esds is used for estimating esds involving l.s. planes.
Refinement. Refinement of F^2^ against ALL reflections. The weighted R-factor wR and goodness of fit S are based on F^2^, conventional R-factors R are based on F, with F set to zero for negative F^2^. The threshold expression of F^2^ > 2sigma(F^2^) is used only for calculating R-factors(gt) etc. and is not relevant to the choice of reflections for refinement. R-factors based on F^2^ are statistically about twice as large as those based on F, and R- factors based on ALL data will be even larger.

### Fractional atomic coordinates and isotropic or equivalent isotropic displacement parameters (Å^2^)

**Table:**

	*x*	*y*	*z*	*U*_iso_*/*U*_eq_	
Cd1	0.5000	0.0000	0.0000	0.02740 (11)	
S1	0.67420 (11)	0.26053 (7)	0.52095 (5)	0.03489 (19)	
N1	0.7260 (3)	0.0299 (2)	0.59906 (16)	0.0269 (5)	
H1A	0.7363	−0.0334	0.6431	0.032*	
N2	0.6614 (4)	0.1938 (2)	0.71990 (17)	0.0357 (6)	
H2A	0.6700	0.1374	0.7693	0.043*	
H2B	0.6361	0.2761	0.7325	0.043*	
O1	0.6235 (3)	0.16660 (17)	0.06762 (15)	0.0342 (5)	
O1W	0.4780 (4)	0.5348 (2)	0.28331 (18)	0.0480 (6)	
H1WA	0.5186	0.4890	0.2317	0.072*	
H1WB	0.3871	0.4986	0.3141	0.072*	
O2	0.6139 (3)	0.35871 (19)	0.14105 (15)	0.0394 (5)	
O2W	0.1911 (4)	0.4499 (3)	0.59727 (19)	0.0624 (7)	
H2WA	0.3036	0.4588	0.6234	0.094*	
H2WB	0.2054	0.4335	0.5340	0.094*	
O3	0.2779 (3)	0.01702 (17)	0.13613 (13)	0.0333 (5)	
O3W	0.1839 (4)	0.3984 (2)	0.37312 (18)	0.0552 (6)	
H3WA	0.0724	0.4490	0.3713	0.083*	
H3WB	0.1880	0.3405	0.3274	0.083*	
O4	0.2247 (3)	0.16445 (18)	0.25914 (14)	0.0342 (5)	
O5	0.2679 (3)	0.16742 (17)	−0.07122 (13)	0.0297 (4)	
C6	0.1262 (4)	0.3800 (3)	−0.1106 (2)	0.0329 (6)	
H6A	0.1338	0.3824	−0.1846	0.039*	
H6B	0.1048	0.4688	−0.0854	0.039*	
C5	−0.0403 (4)	0.3011 (3)	−0.0718 (2)	0.0348 (7)	
H5A	−0.1379	0.3537	−0.0286	0.042*	
H5B	−0.1095	0.2677	−0.1280	0.042*	
C1	0.3151 (4)	0.3013 (2)	−0.06518 (19)	0.0268 (6)	
H1B	0.4400	0.3149	−0.1013	0.032*	
C4	0.0804 (4)	0.1902 (3)	−0.0110 (2)	0.0290 (6)	
H4A	0.0123	0.1123	−0.0029	0.035*	
C2	0.3217 (4)	0.3217 (2)	0.05110 (19)	0.0236 (6)	
H2C	0.2807	0.4145	0.0659	0.028*	
C3	0.1510 (4)	0.2401 (2)	0.08993 (19)	0.0240 (6)	
H3A	0.0406	0.2998	0.1206	0.029*	
C7	0.5339 (4)	0.2791 (3)	0.0913 (2)	0.0264 (6)	
C8	0.2237 (4)	0.1329 (3)	0.1673 (2)	0.0266 (6)	
C9	0.7242 (4)	0.1270 (3)	0.4387 (2)	0.0300 (6)	
C10	0.7405 (4)	0.1277 (3)	0.3330 (2)	0.0395 (7)	
H10A	0.7240	0.2058	0.2956	0.047*	
C11	0.7818 (5)	0.0087 (3)	0.2852 (2)	0.0451 (8)	
H11A	0.7938	0.0065	0.2144	0.054*	
C12	0.8059 (4)	−0.1076 (3)	0.3408 (2)	0.0408 (7)	
H12A	0.8337	−0.1865	0.3065	0.049*	
C13	0.7897 (4)	−0.1093 (3)	0.4460 (2)	0.0327 (6)	
H13A	0.8053	−0.1876	0.4831	0.039*	
C14	0.7494 (4)	0.0096 (3)	0.49408 (19)	0.0257 (6)	
C15	0.6867 (4)	0.1544 (3)	0.6253 (2)	0.0269 (6)	

### Atomic displacement parameters (Å^2^)

**Table:**

	*U*^11^	*U*^22^	*U*^33^	*U*^12^	*U*^13^	*U*^23^
Cd1	0.03353 (18)	0.01935 (16)	0.02779 (17)	0.00173 (11)	0.00364 (12)	−0.00422 (11)
S1	0.0462 (5)	0.0268 (4)	0.0307 (4)	−0.0022 (3)	−0.0007 (3)	0.0056 (3)
N1	0.0304 (13)	0.0259 (12)	0.0248 (12)	−0.0054 (9)	−0.0008 (9)	0.0044 (9)
N2	0.0538 (16)	0.0271 (13)	0.0257 (12)	−0.0046 (11)	0.0014 (11)	−0.0006 (10)
O1	0.0308 (11)	0.0251 (10)	0.0456 (12)	0.0010 (8)	−0.0048 (9)	−0.0076 (9)
O1W	0.0570 (16)	0.0340 (12)	0.0522 (14)	−0.0043 (10)	0.0143 (11)	−0.0108 (10)
O2	0.0370 (12)	0.0364 (12)	0.0467 (12)	−0.0089 (9)	−0.0047 (9)	−0.0161 (10)
O2W	0.0599 (16)	0.0736 (18)	0.0512 (15)	0.0011 (14)	−0.0085 (12)	−0.0075 (14)
O3	0.0461 (12)	0.0223 (10)	0.0289 (10)	0.0031 (8)	0.0099 (9)	0.0026 (8)
O3W	0.0620 (16)	0.0484 (15)	0.0533 (15)	−0.0003 (12)	0.0115 (12)	−0.0161 (12)
O4	0.0501 (13)	0.0284 (10)	0.0235 (10)	−0.0034 (9)	0.0028 (9)	0.0002 (8)
O5	0.0389 (11)	0.0233 (10)	0.0255 (10)	0.0012 (8)	−0.0019 (8)	−0.0045 (8)
C6	0.0436 (18)	0.0274 (15)	0.0262 (14)	0.0003 (12)	−0.0045 (12)	0.0035 (12)
C5	0.0343 (17)	0.0332 (16)	0.0357 (16)	0.0002 (12)	−0.0086 (13)	−0.0007 (13)
C1	0.0333 (15)	0.0227 (14)	0.0240 (14)	−0.0036 (11)	0.0058 (11)	0.0021 (11)
C4	0.0282 (15)	0.0226 (14)	0.0364 (16)	−0.0041 (11)	−0.0021 (12)	−0.0001 (12)
C2	0.0302 (15)	0.0151 (13)	0.0250 (13)	−0.0004 (10)	0.0003 (11)	−0.0035 (10)
C3	0.0250 (14)	0.0203 (13)	0.0254 (13)	0.0009 (10)	0.0052 (11)	−0.0013 (11)
C7	0.0310 (15)	0.0238 (15)	0.0251 (14)	−0.0066 (12)	0.0041 (11)	−0.0012 (11)
C8	0.0243 (14)	0.0258 (15)	0.0295 (15)	−0.0040 (11)	0.0086 (11)	0.0030 (12)
C9	0.0268 (15)	0.0329 (16)	0.0302 (15)	−0.0037 (12)	−0.0033 (11)	0.0037 (12)
C10	0.0404 (18)	0.052 (2)	0.0262 (15)	−0.0056 (14)	−0.0030 (13)	0.0081 (14)
C11	0.0399 (19)	0.072 (2)	0.0240 (15)	−0.0108 (16)	0.0002 (13)	−0.0062 (16)
C12	0.0357 (17)	0.0481 (19)	0.0387 (18)	−0.0045 (14)	0.0006 (13)	−0.0152 (15)
C13	0.0289 (16)	0.0330 (16)	0.0367 (16)	−0.0048 (12)	−0.0021 (12)	−0.0043 (13)
C14	0.0188 (14)	0.0331 (15)	0.0253 (14)	−0.0034 (11)	−0.0022 (10)	−0.0004 (12)
C15	0.0273 (15)	0.0267 (15)	0.0270 (14)	−0.0046 (11)	−0.0025 (11)	0.0020 (12)

### Geometric parameters (Å, º)

**Table:**

Cd1—O1^i^	2.2108 (18)	C6—C1	1.530 (4)
Cd1—O1	2.2108 (18)	C6—C5	1.543 (4)
Cd1—O3	2.2954 (18)	C6—H6A	0.9700
Cd1—O3^i^	2.2954 (18)	C6—H6B	0.9700
Cd1—O5^i^	2.3499 (17)	C5—C4	1.527 (4)
Cd1—O5	2.3499 (17)	C5—H5A	0.9700
S1—C15	1.733 (3)	C5—H5B	0.9700
S1—C9	1.754 (3)	C1—C2	1.543 (3)
N1—C15	1.324 (3)	C1—H1B	0.9800
N1—C14	1.397 (3)	C4—C3	1.532 (4)
N1—H1A	0.8600	C4—H4A	0.9800
N2—C15	1.311 (3)	C2—C7	1.528 (4)
N2—H2A	0.8600	C2—C3	1.583 (3)
N2—H2B	0.8600	C2—H2C	0.9800
O1—C7	1.271 (3)	C3—C8	1.522 (3)
O1W—H1WA	0.8501	C3—H3A	0.9800
O1W—H1WB	0.8500	C9—C10	1.388 (4)
O2—C7	1.240 (3)	C9—C14	1.391 (4)
O2W—H2WA	0.8499	C10—C11	1.378 (4)
O2W—H2WB	0.8500	C10—H10A	0.9300
O3—C8	1.271 (3)	C11—C12	1.384 (4)
O3W—H3WA	0.8500	C11—H11A	0.9300
O3W—H3WB	0.8500	C12—C13	1.381 (4)
O4—C8	1.252 (3)	C12—H12A	0.9300
O5—C1	1.461 (3)	C13—C14	1.378 (4)
O5—C4	1.464 (3)	C13—H13A	0.9300
			
O1^i^—Cd1—O1	180.00 (10)	C2—C1—H1B	113.6
O1^i^—Cd1—O3	94.23 (7)	O5—C4—C5	101.6 (2)
O1—Cd1—O3	85.77 (7)	O5—C4—C3	102.5 (2)
O1^i^—Cd1—O3^i^	85.77 (7)	C5—C4—C3	110.9 (2)
O1—Cd1—O3^i^	94.23 (7)	O5—C4—H4A	113.6
O3—Cd1—O3^i^	180.00 (6)	C5—C4—H4A	113.6
O1^i^—Cd1—O5^i^	82.90 (6)	C3—C4—H4A	113.6
O1—Cd1—O5^i^	97.10 (6)	C7—C2—C1	110.9 (2)
O3—Cd1—O5^i^	96.23 (6)	C7—C2—C3	116.9 (2)
O3^i^—Cd1—O5^i^	83.77 (6)	C1—C2—C3	100.8 (2)
O1^i^—Cd1—O5	97.10 (6)	C7—C2—H2C	109.3
O1—Cd1—O5	82.90 (6)	C1—C2—H2C	109.3
O3—Cd1—O5	83.77 (6)	C3—C2—H2C	109.3
O3^i^—Cd1—O5	96.23 (6)	C8—C3—C4	114.5 (2)
O5^i^—Cd1—O5	180.00 (8)	C8—C3—C2	113.7 (2)
C15—S1—C9	90.15 (13)	C4—C3—C2	101.3 (2)
C15—N1—C14	114.6 (2)	C8—C3—H3A	109.0
C15—N1—H1A	122.7	C4—C3—H3A	109.0
C14—N1—H1A	122.7	C2—C3—H3A	109.0
C15—N2—H2A	120.0	O2—C7—O1	123.2 (3)
C15—N2—H2B	120.0	O2—C7—C2	118.2 (2)
H2A—N2—H2B	120.0	O1—C7—C2	118.5 (2)
C7—O1—Cd1	129.36 (17)	O4—C8—O3	123.6 (2)
H1WA—O1W—H1WB	108.2	O4—C8—C3	117.4 (2)
H2WA—O2W—H2WB	110.7	O3—C8—C3	119.0 (2)
C8—O3—Cd1	115.11 (15)	C10—C9—C14	120.7 (3)
H3WA—O3W—H3WB	110.4	C10—C9—S1	128.6 (2)
C1—O5—C4	95.87 (18)	C14—C9—S1	110.64 (19)
C1—O5—Cd1	117.20 (15)	C11—C10—C9	117.7 (3)
C4—O5—Cd1	112.02 (14)	C11—C10—H10A	121.1
C1—C6—C5	101.8 (2)	C9—C10—H10A	121.1
C1—C6—H6A	111.4	C10—C11—C12	121.2 (3)
C5—C6—H6A	111.4	C10—C11—H11A	119.4
C1—C6—H6B	111.4	C12—C11—H11A	119.4
C5—C6—H6B	111.4	C13—C12—C11	121.5 (3)
H6A—C6—H6B	109.3	C13—C12—H12A	119.3
C4—C5—C6	102.0 (2)	C11—C12—H12A	119.3
C4—C5—H5A	111.4	C14—C13—C12	117.5 (3)
C6—C5—H5A	111.4	C14—C13—H13A	121.2
C4—C5—H5B	111.4	C12—C13—H13A	121.2
C6—C5—H5B	111.4	C13—C14—C9	121.4 (2)
H5A—C5—H5B	109.2	C13—C14—N1	126.8 (2)
O5—C1—C6	101.6 (2)	C9—C14—N1	111.9 (2)
O5—C1—C2	102.46 (18)	N2—C15—N1	124.0 (2)
C6—C1—C2	110.8 (2)	N2—C15—S1	123.3 (2)
O5—C1—H1B	113.6	N1—C15—S1	112.69 (19)
C6—C1—H1B	113.6		
			
O3—Cd1—O1—C7	−55.7 (2)	C5—C4—C3—C2	−72.4 (2)
O3^i^—Cd1—O1—C7	124.3 (2)	C7—C2—C3—C8	2.8 (3)
O5^i^—Cd1—O1—C7	−151.4 (2)	C1—C2—C3—C8	123.0 (2)
O5—Cd1—O1—C7	28.6 (2)	C7—C2—C3—C4	−120.5 (2)
O1^i^—Cd1—O3—C8	−146.03 (18)	C1—C2—C3—C4	−0.3 (2)
O1—Cd1—O3—C8	33.97 (18)	Cd1—O1—C7—O2	170.25 (19)
O5^i^—Cd1—O3—C8	130.68 (18)	Cd1—O1—C7—C2	−13.6 (3)
O5—Cd1—O3—C8	−49.32 (18)	C1—C2—C7—O2	126.0 (2)
O1^i^—Cd1—O5—C1	−167.19 (16)	C3—C2—C7—O2	−119.3 (3)
O1—Cd1—O5—C1	12.81 (16)	C1—C2—C7—O1	−50.4 (3)
O3—Cd1—O5—C1	99.31 (16)	C3—C2—C7—O1	64.3 (3)
O3^i^—Cd1—O5—C1	−80.69 (16)	Cd1—O3—C8—O4	−131.4 (2)
O1^i^—Cd1—O5—C4	83.43 (16)	Cd1—O3—C8—C3	49.3 (3)
O1—Cd1—O5—C4	−96.57 (16)	C4—C3—C8—O4	−158.7 (2)
O3—Cd1—O5—C4	−10.07 (15)	C2—C3—C8—O4	85.5 (3)
O3^i^—Cd1—O5—C4	169.93 (15)	C4—C3—C8—O3	20.6 (3)
C1—C6—C5—C4	−0.2 (3)	C2—C3—C8—O3	−95.1 (3)
C4—O5—C1—C6	−57.3 (2)	C15—S1—C9—C10	179.8 (3)
Cd1—O5—C1—C6	−175.78 (14)	C15—S1—C9—C14	−0.5 (2)
C4—O5—C1—C2	57.3 (2)	C14—C9—C10—C11	0.1 (4)
Cd1—O5—C1—C2	−61.1 (2)	S1—C9—C10—C11	179.8 (2)
C5—C6—C1—O5	35.4 (2)	C9—C10—C11—C12	0.2 (5)
C5—C6—C1—C2	−72.9 (3)	C10—C11—C12—C13	0.0 (5)
C1—O5—C4—C5	57.2 (2)	C11—C12—C13—C14	−0.3 (4)
Cd1—O5—C4—C5	179.65 (15)	C12—C13—C14—C9	0.6 (4)
C1—O5—C4—C3	−57.6 (2)	C12—C13—C14—N1	179.6 (2)
Cd1—O5—C4—C3	64.89 (19)	C10—C9—C14—C13	−0.5 (4)
C6—C5—C4—O5	−34.9 (3)	S1—C9—C14—C13	179.8 (2)
C6—C5—C4—C3	73.4 (3)	C10—C9—C14—N1	−179.6 (2)
O5—C1—C2—C7	89.5 (2)	S1—C9—C14—N1	0.7 (3)
C6—C1—C2—C7	−162.8 (2)	C15—N1—C14—C13	−179.6 (3)
O5—C1—C2—C3	−34.9 (2)	C15—N1—C14—C9	−0.5 (3)
C6—C1—C2—C3	72.8 (2)	C14—N1—C15—N2	−179.4 (2)
O5—C4—C3—C8	−87.4 (2)	C14—N1—C15—S1	0.1 (3)
C5—C4—C3—C8	164.8 (2)	C9—S1—C15—N2	179.7 (2)
O5—C4—C3—C2	35.3 (2)	C9—S1—C15—N1	0.2 (2)

Symmetry code: (i) −*x*+1, −*y*, −*z*.

### Hydrogen-bond geometry (Å, º)

**Table:**

*D*—H···*A*	*D*—H	H···*A*	*D*···*A*	*D*—H···*A*
O1*W*—H1*WA*···O2	0.85	1.85	2.682 (3)	167
O2*W*—H2*WB*···O3*W*	0.85	2.15	2.995 (3)	170
O1*W*—H1*WB*···O3*W*	0.85	1.96	2.806 (3)	173
O3*W*—H3*WB*···O4	0.85	2.02	2.837 (3)	161
N1—H1*A*···O4^ii^	0.86	1.84	2.700 (3)	176
N2—H2*A*···O3^ii^	0.86	2.00	2.845 (3)	169
N2—H2*B*···O1*W*^iii^	0.86	2.00	2.824 (3)	160
O2*W*—H2*WA*···O1*W*^iii^	0.85	1.93	2.758 (4)	164
O3*W*—H3*WA*···O2*W*^iv^	0.85	1.96	2.794 (3)	166

Symmetry codes: (ii) −*x*+1, −*y*, −*z*+1; (iii) −*x*+1, −*y*+1, −*z*+1; (iv) −*x*, −*y*+1, −*z*+1.
